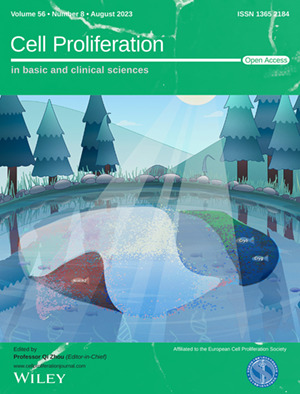# Featured Cover

**DOI:** 10.1111/cpr.13532

**Published:** 2023-08-01

**Authors:** Yunqian Yao, Ling Wei, Zhenhua Chen, Hao Li, Jiao Qi, Qingfeng Wu, Xingtao Zhou, Yi Lu, Xiangjia Zhu

## Abstract

The cover image is based on the Original Article *Single‐cell RNA sequencing: Inhibited Notch2 signalling underlying the increased lens fibre cells differentiation in high myopia* by Yunqian Yao et al., https://doi.org/10.1111/cpr.13412.